# Supplementary material for: Regulator of G-Protein Signalling 9: A New Candidate Gene for Sweet Food Liking?
Source: Foods. 2023 Apr 22;12(9):1739. doi: 10.3390/foods12091739 (PMC10178705; doi:10.3390/foods12091739)
Supplement: Supplementary file 1 [file foods-12-01739-s001.zip › Supplementary_tables_1_and4.pdf]

Table S1. Mean and standard deviation of the liking for sweet foods included in the group.

Liking	Discovery sample		Replication sample
	VBI	CAR	FVG
Ice cream	7.7 (1.7)	7.6 (1.8)	7.7 (1.6)
Panettone	6.3 (2.2)	6.5 (2.5)	6.2 (2.2)
Whipped cream	6.1 (2.6)	5.5 (3.0)	5.8 (2.7)
Milk chocolate	6.6 (2.4)	6.6 (2.7)	7.0 (2.1)
Marzipan	4.4 (2.8)	5.2 (3.0)	4.7 (2.8)
Biscuits	6.9 (1.6)	Not available	6.5 (2.0)
Cake	7.2 (1.7)	Not available	6.9 (2.0)
Marmalade	7.0 (1.8)	Not available	6.7 (2.1)
Nutella	6.6 (2.4)	Not available	6.1 (2.7)
Cake icing	4.6 (2.6)	Not available	4.5 (2.7)
Hot chocolate with cream	6.3 (2.5)	Not available	6.0 (2.7)

CAR=Carlantino, VBI=Val Borbera, FVG=Friuli Venezia Giulia.

Table S4. Results of the regression analysis on the individual food liking in the combined sample of the discovery and replication to verify the association with the rs58931966 SNP of the *RGS9* gene.

**Trait**	**Population**	**SNP**	**Gene**	**EA/OA**	**N**	**Effect**	**StdErr**	**P-value**
Ice cream	CAR+VBI+FVG	rs58931966	*RGS9*	A/T	2514	-0.434	0.141	0.0021
Panettone	CAR+VBI+FVG	rs58931966	*RGS9*	A/T	2509	-0.377	0.073	$2.52x10^{-7}$
Whipped cream	CAR+VBI+FVG	rs58931966	*RGS9*	A/T	2442	-0.322	0.089	$3.0x10^{-4}$
Milk chocolate	CAR+VBI+FVG	rs58931966	*RGS9*	A/T	1401	-0.263	0.106	0.0133
Marzipan	CAR+VBI+FVG	rs58931966	*RGS9*	A/T	1054	-0.429	0.138	0.0019
Biscuits	VBI+FVG	rs58931966	*RGS9*	A/T	1074	-0.191	0.094	0.0422
Cake	VBI+FVG	rs58931966	*RGS9*	A/T	1073	-0.259	0.096	0.0073
Marmalade	VBI+FVG	rs58931966	*RGS9*	A/T	1074	-0.172	0.099	0.0843
Nutella	VBI+FVG	rs58931966	*RGS9*	A/T	1073	-0.371	0.131	0.0046
Cake icing	VBI+FVG	rs58931966	*RGS9*	A/T	1062	-0.487	0.137	0.0004
Hot chocolate with cream	VBI+FVG	rs58931966	*RGS9*	A/T	1060	-0.372	0.135	0.0059

EA=effect allele, OA=other allele; *CAR=Carlantino, VBI=Val Borbera, FVG=Friuli Venezia Giulia.*
