# Supplementary figures and images for: Regulator of G-Protein Signalling 9: A New Candidate Gene for Sweet Food Liking?
Source: Foods. 2023 Apr 22;12(9):1739. doi: 10.3390/foods12091739 (PMC10178705; doi:10.3390/foods12091739)

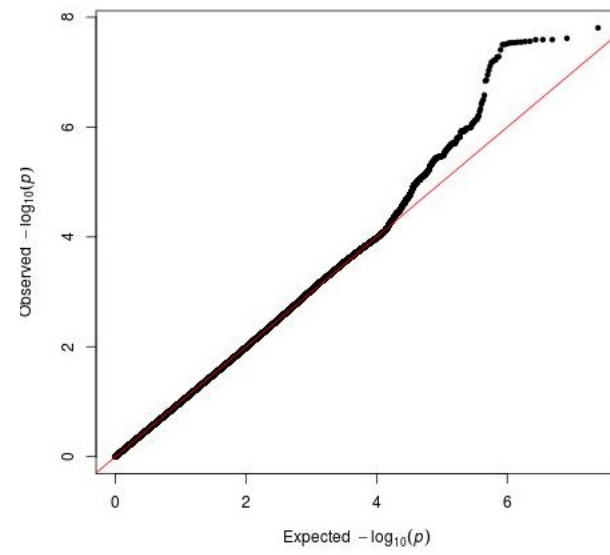

Supplement: Supplementary file 1 [file foods-12-01739-s001.zip › Figure S1 QQ-plot of the meta-analysis on sweet food liking on CAR and VBI.pdf]
